# High-Temperature Hydrogen Sensing Performance of Ni-Doped TiO_2_ Prepared by Co-Precipitation Method

**DOI:** 10.3390/s20215992

**Published:** 2020-10-22

**Authors:** Roussin Lontio Fomekong, Klemens Kelm, Bilge Saruhan

**Affiliations:** 1 Higher Teacher Training College, University of Yaounde I, Yaounde P.O. BOX 47, Cameroon; lonforou@yahoo.fr; 2 German Aerospace Center (DLR), Institute of Materials Research, 51147 Cologne, Germany; klemens.kelm@dlr.de

**Keywords:** high-temperature sensing, Ni-doped TiO_2_, hydrogen, gas sensor, co-precipitation

## Abstract

This work deals with the substantially high-temperature hydrogen sensors required by combustion and processing technologies. It reports the synthesis of undoped and Ni-doped TiO_2_ (with 0, 0.5, 1 and 2 mol.% of Ni) nanoparticles by a co-precipitation method and the obtained characteristics applicable for this purpose. The effect of nickel doping on the morphological variation, as well as on the phase transition from anatase to rutile, of TiO_2_ was investigated by scanning electron microscopy, X-ray diffraction and Raman spectroscopy. The resistive sensors prepared with these powders were tested toward H_2_ at 600 °C. The results indicate that 0.5% Ni-doped TiO_2_ with almost equal amounts of anatase and rutile shows the best H_2_ sensor response (ΔR/R0 = 72%), response rate and selectivity. The significant improvement of the sensing performance of 0.5% Ni-doped TiO_2_ is mainly attributed to the formation of the highest number of n-n junctions present between anatase and rutile, which influence the quantity of adsorbed oxygen (i.e., the active reaction site) on the surface and the conductivity of the material.

## 1. Introduction

Many manufacturing industries, such as those of steel, metals and semiconductors, use hydrogen in their processes. In the electronic and metallurgical plants, hydrogen is used as a reducing agent, while it is used as a carrier gas in gas chromatography. Hydrogen is also considered as a building block for the production of ammonia, a substance necessary in many chemical companies. Nowadays, hydrogen is the best candidate to replace the hydrocarbon-based fuels used in many combustion engines such as those in automobiles and aircraft, which are responsible for much of today’s air pollution [1,2,3]. Hydrogen seems to be a green, renewable energy carrier that can help solve the problems of non-sustainable energy use (fossil fuels). However, the efficient application of hydrogen requires careful consideration of the relevant safety concern. In fact, its physico-chemical properties (low boiling point, high burning velocity, low ignition energy and high diffusivity) make hydrogen a highly explosive gas [4,5]. Moreover, as hydrogen is colorless, odorless and tasteless, the ability to detect a hydrogen leak by means of selective sensors is highly desired [6]. In addition, hydrogen production through a solid oxide electrolyzer cell requires high-temperature electrolysis of water (500–850 °C) and thus, needs rapid and sensitive hydrogen sensors that can operate in harsh and complex environments (T > 400 °C, in the presence of many interfering gases) enabling real-time gas leakage control [7]. Sensing devices for real-time H_2_-monitoring should be able to function with good stability and deliver reproducible data under harsh environmental conditions containing highly reducing and/or oxidizing gas streams at high gas temperatures of 500−700 °C [7].

Among resistive sensors, semiconducting metal oxides are attracting more attention as gas sensing materials due to their simple working principle, yielding high sensitivity and being lightweight and low cost [8,9]. The literature reports that optimum sensitivity for this sensor family is obtained at temperatures below 400 °C for instance with CuO, SnO_2_, ZnO and WO_3_ [10,11,12,13,14]. On the other hand, TiO_2_ is capable of operating as a gas-sensing material at higher temperatures (T > 400 °C) [15,16,17]. TiO_2_ is easy to manufacture, non-toxic and a chemically stable semiconducting metal oxide, which is already finding applications in many areas [18]. However, the high resistivity of n-type semiconductor TiO_2_ constitutes a serious limitation for its application as a promising sensing material [19,20]. TiO_2_ has two polymorphs: anatase and rutile. Rutile is the less active polymorph and has a bandgap of Eg ≈ 3 eV. Whereas anatase, being the more active polymorph of TiO_2_, has a wide bandgap (Eg ≈ 3.2 eV), which is generally responsible for increasing the resistance of electronic components in operation. Therefore, anatase TiO_2_ seems to be the less suitable candidate for wide use as a high-temperature H_2_ sensor. Previous studies have reported that this disadvantage can be overcome by suitably doping the TiO_2_ [21,22,23,24,25,26]. Another main drawback of a resistive semiconductor metal oxide-based sensor such as TiO_2_ is selectivity. In order to enhance the sensor selectivity, one can design hetero-structures with p-n, or n-n junction, dope the materials, use a filter layer, etc. [27,28,29].

Some works in literature report on the high-temperature detection of H_2_. For instance, gallium nitride, nickel-cobalt oxide, nickel oxide, stabilized zirconia and undoped titanium dioxide have all been used for H_2_ detection between 450 and 600 °C [4,15,17,24,30,31,32]. The use of undoped TiO_2_ for high temperature hydrogen detection mentioned in the literature nowadays reports 500 °C as the maximum achievable operating temperature for hydrogen detection. It is already known that doping is an effective way to improve the gas-sensing properties of sensors based on metal oxide semiconductors. Several works report the effect of Ni doping on TiO_2_ sensing properties at low temperatures (<350 °C) [22,25,33]. In fact, substituting Ti by Ni, depending on the synthesis method, could change the conductivity type, inducing effective reduction in the bandgap of anatase titanium dioxide. The presence of Ni as an impurity in the TiO_2_ lattice is also known to create more oxygen vacancies since it induces impurity level. Relying on these, we explored a series of Ni-doped TiO_2_ prepared by the co-precipitation synthesis route and, herein report, to the best of our knowledge for the first time, the high-temperature (600 °C) H_2_ sensing performance achieved with Ni-doping of TiO_2_. The results of this work show that Ni-doping promotes the anatase-to-rutile transition and at a specific content of nickel dopant, the Ni-doped TiO_2_ sensor yields the best sensor performance regarding sensor response, sensor rate, selectivity. A reasonable explanation for the observed enhanced high-temperature H_2_ gas sensing performance of Ni-doped TiO_2_-based semiconductor sensors has been proposed.

## 2. Materials and Methods

### 2.1. Synthesis of Undoped and Ni-Doped TiO_2_ Powders

Undoped and Ni-doped TiO_2_ nanoparticles were prepared using the co-precipitation synthesis route followed by calcination. The starting precursor solutions were first prepared by dissolving nickel acetate in acetic acid while pure ethanol was used separately to dissolve titanium iso-propoxide (TTIP). The adjustment between the previously prepared nickel and titanium solution has been performed so that the final obtained mix would contain 0.0, 0.5, 1.0 and 2.0 mol.% of nickel dopant in TiO_2_ and were labeled as TN0, TN05, TN1 and TN2, respectively. The solutions were then mixed and stirred for 5 min. Oxalic acid was used as the precipitating agent. It was dissolved in absolute ethanol solution and poured progressively into the previously mixed solutions. In order to achieve a total precipitation, the resulting mixtures were stirred for 1 h at room temperature, followed by the filtration and drying of the obtained precipitate at 80 °C. A white powder was obtained for the undoped precursor and a yellowish colored powder for the Ni-doped precursor samples. The prepared precursor powders were then calcined in a muffle furnace under static air for 3 h at 700 °C to obtain the nano-particulate powders.

### 2.2. Material Characterization

For phase analyses, XRD measurements of the prepared samples were carried out on D5000 Siemens Kristalloflex θ–2θ powder diffractometer provided with Bragg–Brentano geometry. The program EVA from BRUKER AXS was used to assign the reflections from the Joint Committee on Powder Diffraction Standards (JCPDS) database to the experimental diffractograms. The Spurr and Myers formula was used to calculate the quantity of anatase and rutile [23]. For this calculation, the individual reflection of anatase (101) and rutile (110) were fitted using Bruker Topas 4.0 software, utilizing a pseudo-Voigt profile function and a 5th order polynomial background correction. The intensities derived from the (101)_anatase_ and (110)_rutile_ reflection were extracted and the ratio was calculated. The lattice parameters were obtained by making a Pawley-fit (Bruker Topas 4.0 software) using a 5th order background polynomial, an intensity correction for the automatic divergence slit and applying a pseudo-Voigt profile function.

The Raman spectra presented here were recorded at room temperature on Bruker Senterra Raman spectrometer (Bruker Optik GmbH, Ettlingen, Germany) under 532 nm and 0.2 mW power laser excitation, which was focused on samples by means of a 50× objective (Olympus MPlan N 50×/0.75).

A Zeiss Ultra 55 Scanning Electron Microscope (SEM) was used to determine the morphology of the particles. This microscope (from Zeiss, Jena, Germany) is fitted out with an energy-dispersive spectrometer X-ray (from Oxford Instruments, Oxford, England) used for semi-quantitative elemental analysis. For this, the experiments were performed at 15 keV with a working distance (distance between the specimen and the lower pole piece in SEM system) of 8 mm. A probe current of 1 nA and acquisition time of 300 s were used to collect the chemical spectra. Integrated Aztec software (AZtec 4.1 SP1, Oxford, England) was used for quantitative analysis of the atomic elements.

Micrometrics Tristar 3000 equipment utilizing N_2_ adsorption at 77.150 K was used to determine the specific surface area of the samples. The samples were degassed under vacuum at 120 °C for 2 h prior to the surface area measurements. The specific surface area was calculated using the Brunauer–Emmet–Teller (BET) equation.

### 2.3. Gas Sensing Tests

For gas-sensing measurements, the prepared undoped TiO_2_ and Ni-doped TiO_2_ powders were deposited as thick films (~20 µm) on alumina substrates fitted with interdigitated Pt-electrodes (the distance between electrode and sensor area is 300 µm), using a simple drop-coating of each corresponding ink. In order to ensure almost the same thickness across the entire sensor, two drops of the corresponding ink were used for each deposition. For the ink preparation, metal oxide powder was mixed vigorously with distilled water (in the powder to water ratio of 1/10). The baseline resistance was stabilized by heating the sensor at 700 °C for 1 h under air flow before starting with the sensor tests. The sensor measurements were performed in dry synthetic air in a specially constructed apparatus consisting of a tube furnace and a custom-built quartz glass reactor providing a thermocouple directed at the sensor. The Keithley 2635A Sourcemeter was used to perform the electrical measurements. The gas concentration was calibrated by an eight-channel mass flow controller from MKS Instruments GmbH (MFC-647b). A flow rate of 400 sccm, a constant current of 1 × 10 ^−6^ A and a voltage of 1 V were fixed for measurement. As undoped and Ni-doped TiO_2_ are n-type semiconductors, the sensor response for n-type semiconductors is defined by (R_gas_/R_air_ – 1) × 100 and (R_air_/R_gas_ – 1) × 100 for oxidizing and reducing gases, respectively.

## 3. Results and Discussion

**Figure 1:**
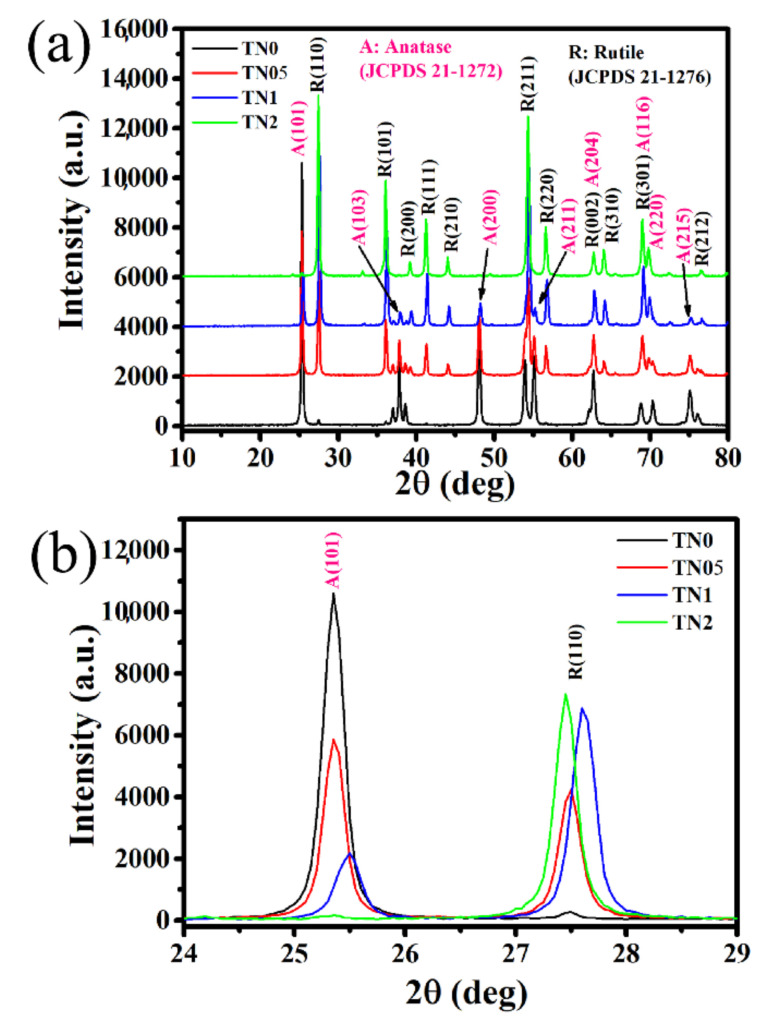
(**a**) Powder XRD patterns of undoped and Ni-doped TiO_2_, (**b**) zoom in on main anatase and rutile peaks.

XRD was used to investigate the phase compositions of all samples after synthesis and calcination at 700 °C. The results of this investigation are given in Figure 1a,b, indicating that anatase is present in those from undoped to 1% Ni-doped TiO_2_, while rutile is the only phase present in the 2% Ni-doped TiO_2_ powders. As Table 1 shows, the amount of anatase decreases as the amount of nickel increases. The amount of anatase (JCPDS 21-1272) is 98, 53, 21 and 1% while the amount of rutile (JCPDS 21-1276) is 2, 47, 79 and 99% for TN0, TN05, TN1 and TN2, respectively. A trace amount of ilmenite, NiTiO_3_ is observed at 32.5° in the TN1 and TN2 samples (JCPDS 33-0960). These results indicate that Ni promotes the transition of anatase to rutile. This is certainly due to the substitution of Ti^4+^ by Ni^3+^. As Figure 1b displays, a shift of the 2θ angles towards the higher values is observed indicating the decrease in the TiO_2_-lattice parameter due to increased Ni-addition. For the anatase polymorph, the lattice parameter a is found as 3.7874, 3.7839 and 3.7836 Å for TN0, TN05 and TN1, respectively (see Table 1). Due to the low amount of anatase in TN2, it was not possible through this investigation to obtain the reliable lattice parameter. It should be mentioned that the ionic radius of Ni^3+^ is smaller than that of Ti^4+^ (Ni^3+^ = 0.700 Å, Ti^4+^ = 0.745 Å). As known, the substitution of Ti^4+^ by cations of small radii and low valence (lesser than four) accelerates the transition to rutile due to the increase in concentration of oxygen vacancies. This can be explained by the fact that the neutrality of the charge resulting from this substitution requires an increase in the level of oxygen vacancies, which in turn leads to the reorganization of chemical bonds to form rutile. On the other hand, the contraction of the cell volume due to the substitution of Ti^4+^ sites by Ni^3+^ leads to a volume reduction in the TiO_2_-lattice and promotes the conversion from anatase to rutile [34].

**Figure 2:**
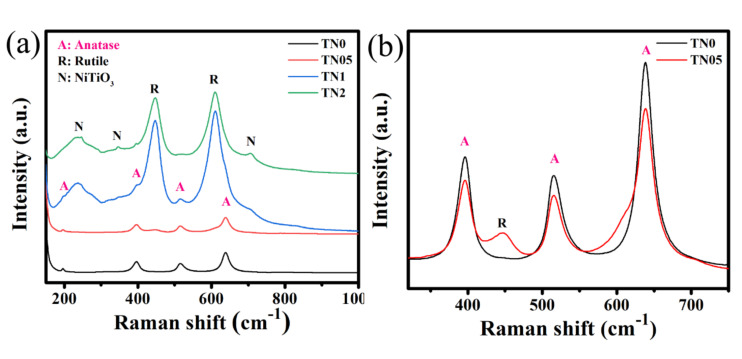
(**a**) Raman spectra of undoped and Ni-doped TiO_2_, (**b**) zoom in on main peaks of TN0 and TN05.

The Raman spectra of the samples were obtained between the wavenumbers of 175–800 cm^−1^. The results, which are presented in Figure 2, show that the main Raman signals came from TiO_2_. The samples TN0 and TN05 show very strong Raman signals, with peaks at 196(Eg), 396(B1g), 517(A1g) and 638(B1g) cm^−1^ from the typical anatase TiO_2_ phase (see Figure 2a) [35]. A weak peak at 447 cm^−1^ (Eg) attributed to rutile is observed in TN05. Samples TN1 and TN2, which contain larger amounts of Ni, present the Raman signals corresponding to both the anatase and rutile (447(Eg), 612(A1g) cm^−1^) phase [36]. The peak intensities of anatase are weak in TN1 while almost absent in TN2. In addition to the anatase and rutile phases, another set of Raman vibrations emerges in the TN1 and TN2 samples. The peaks at 244, 345 and 706 cm^−1^ are assigned to a trace amount of ilmenite, NiTiO_3_ [37]. These results are in accordance with the obtained XRD results. For TN0 and TN05, where anatase is the main phase, the peak intensities decrease with the presence of Ni (Figure 2b) and for TN1 and TN2 where rutile is the main phase, the peak intensities also decrease where the amount of Ni is high (e.g., TN2). This decrease in observed intensities in the Raman spectra may be assigned to the raise of oxygen vacancies. In fact, during the substitution of Ti^4+^ by Ni^3+^, the oxygen vacancies concentration can increase due to the balancing of charge neutrality, depending on the difference between their ionic charges. Due to these vacancies, it is likely that the lattice distortion can occur, thus causing a change in the Raman intensity, as previously reported by Chanda et al. [35].

**Figure 3:**
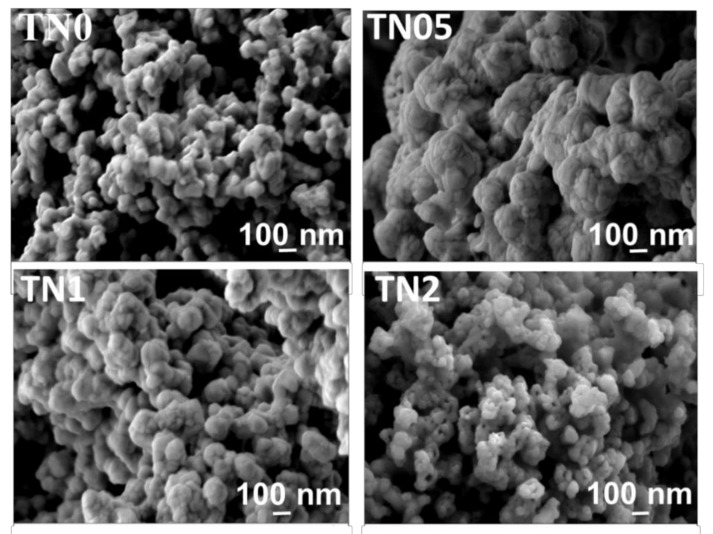
SEM images of undoped and all Ni-doped TiO_2_ powders prepared by the co-precipitation route.

Figure 3 shows the morphology of the synthesized powders investigated by SEM. As far as the undoped powder sample is concerned, the microstructural investigation reveals spherical nanoparticles with sizes around 70 nm, which tend to agglomerate. Whereas a significant influence of Ni doping and dopant content on powder morphology was observed. While the TN05 sample shows more agglomerated spherical particles, the TN1 and TN2 samples show less agglomeration with the appearance of small pores and particle size reduction for the TN2 sample. The results of the semi-quantitative analysis performed by Energy-Dispersive X-ray (EDX) on TN0, TN05, TN1 and TN2 are summarized in Table 2. The Ni/Ti atomic ratio percentages were 0, 0.48, 0.97, 1.98, respectively (cf. 0, 0.5, 1 and 2 expected). These results indicate that the expected composition was obtained, confirming the efficiency of the applied co-precipitation synthesis route.

BET measurements indicated that the specific surface areas of the synthesized samples were found as 7.8, 6.9, 6.4 and 10.7 m^2^/g for TN0, TN05, TN1 and TN2, respectively (Table 1). The small surface area observed is explained by the tendency of particles to agglomerate, which limit the gas adsorption on the material surface. The surface area is thus almost constant for the undoped TiO_2_, 0.5% Ni-doped TiO_2_ and 1% Ni-doped TiO_2_ but increases for 2% Ni-doped TiO_2_. This increase is certainly due to the decrease in particle size and the presence of small pores as revealed by SEM, which may enhance the N_2_ gas adsorption.

Based on our previous results on undoped and Al, Cr and Co-doped TiO_2_, 600 °C was chosen as the optimum sensing temperature in this work [21,23]. In fact, both the sensor signal and response time decrease when the temperature increases. A balance is therefore found at 600 °C. The operating temperature was also kept below 700 °C to avoid changes in the TiO_2_ polymorph, which generally occur above this temperature and would influence the study of the effect of Ni-dopant on the sensing properties. The sensing properties of the undoped and doped TiO_2_ with different Ni-contents to 10,000 ppm H_2_ were investigated at 600 °C using dry synthetic air as the carrier gas.

**Figure 4:**
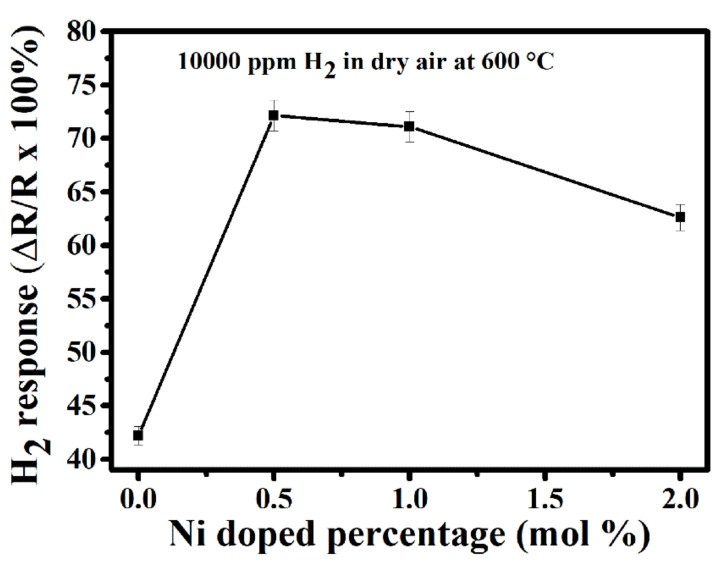
Response of gas sensors based on the co-precipitation synthesized undoped TiO_2_ and on all the Ni-doped TiO_2_ to 10,000 ppm of hydrogen gas at the optimum operating temperature of 600 °C.

**Figure 5:**
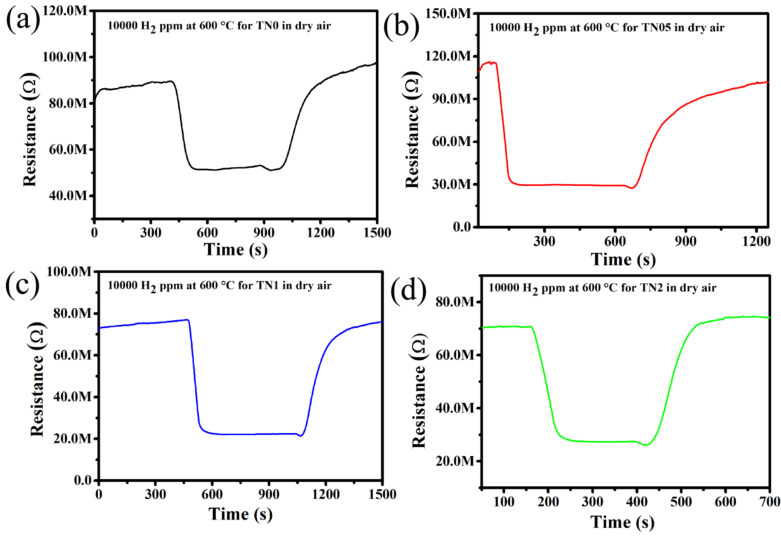
Dynamic response toward 10,000 ppm H_2_ at 600 °C for (**a**) undoped TiO_2_, (**b**) 0.5% Ni-doped TiO2-TN05, (**c**) 1.0% Ni-doped TiO2-TN1 and (**d**) 2.0% Ni-doped TiO2-TN2.

The responses of the undoped TiO_2_ and all Ni-doped TiO_2_ towards 10,000 ppm H_2_ in dry synthetic air at 600 °C are shown in Figure 4. The sensor responses are 42, 72, 70 and 62% for TN0, TN05, TN1 and TN2, respectively. It can be observed that the sensor response increases greatly as Ni-content increases up to 0.5 mol.% and then decreases slowly with further increase in the Ni-content to 2.0 mol.%. This implies that the sensor reaches its maximum response of 72% with 0.5 mol.% of Ni dopant. It can be assumed that this enhancement of gas sensor response may be due to the formation of a n-n junction between the anatase (Eg = 3.2 eV) and rutile (Eg = 3.0 eV) phases. As revealed by XRD results given in Table 1, the TN05 sample contains almost the same amount of anatase and rutile phases (which is not the case with the other samples in this work), and thus, the highest amount of n-n junctions are expected to be present in this sample. Relying on this fact and the above assumption, it is reasonable to conclude that the optimum sensor response observed with TN05 is due to the presence of larger n-n junctions. The presence of a maximum number of n-n junctions in TN05 was confirmed by the dynamic response to H_2_ at 600 °C (with time interval of H_2_ of 8 min), which is given in Figure 5 for the four sensors where the baseline resistance of the TN05 sample (R_air_ = 116 MΩ) in air is higher than that of the TN0 (R_air_ = 89 MΩ), TN1 (R_air_ = 77 MΩ) and TN2 (R_air_ = 70 MΩ) samples. In fact, as reported in literature [38,39], the presence of a junction between particles with different band gaps provokes the increase in baseline resistance. This means the more junction is present, the higher the baseline resistance gets. This kind of junction effect has also been reported for other n-n junction systems such as ZnO-SnO_2_ [38] and SnO_2_-WO_3_ [39]. A detailed explanation of this phenomenon is given in the section “sensing mechanism”.

**Figure 6:**
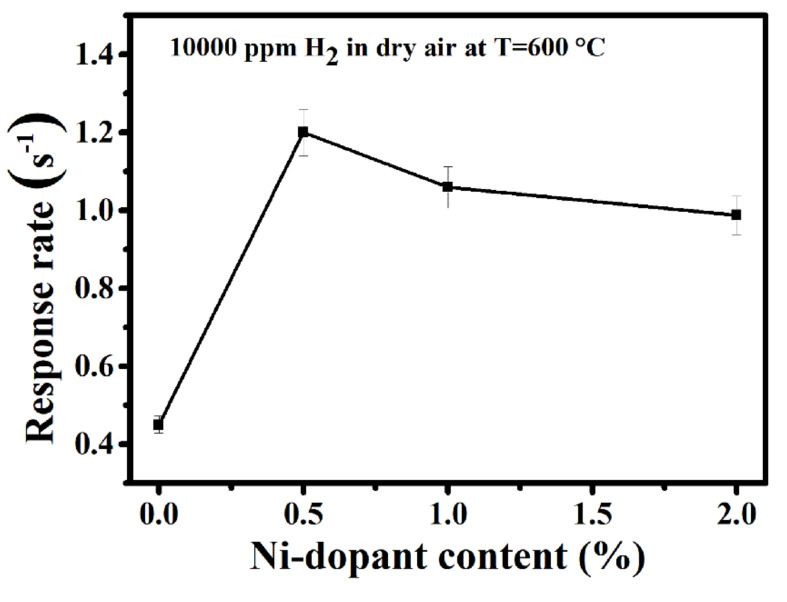
Response rate of undoped TiO_2_ and all Ni-doped TiO_2_ toward 10,000 ppm H_2_ in dry air at 600 °C.

For the detection of hydrogen leakage, the response rate is a very important parameter, as the sensor must detect the leak as soon as possible to avoid an explosion. We therefore investigated the effect of Ni-doping of TiO_2_ on the hydrogen response rate in dry air. The sensor response rate was defined by employing the average rate change of sensor response during the response processes. For this, 90% of the full sensor response was divided by the response times (0.9 S/t_res_). As revealed by Figure 6, the response rates are 0.45, 1.2, 1.06 and 0.98 for TN0, TN05, TN1 and TN2, respectively. It was observed that, in general, doping with Ni increases the response rate by at least a factor of two, and the maximum increase is obtained for TN05. This means, for 0.5 mol.% of Ni (TN05), the sensor is taking less time to reach the relevant resistance value in the presence of H_2_ and can therefore detect a H_2_ leak rapidly enough. This enhancement in the response rate may be due to the formation of n-n junctions, which could speed up the formation of ionized oxygen species (e.g., O^2−^) on the sensor surface during the detection process.

Based on the fact that the n-n junction may have some interesting effects on TN05 for the detection of H_2_ at high temperature, further investigations were carried out on the gas sensor properties of this material to study the effect of operating temperature on sensor response and on selectivity, and the effect of H_2_ concentration on sensor response and recovery times.

**Figure 7:**
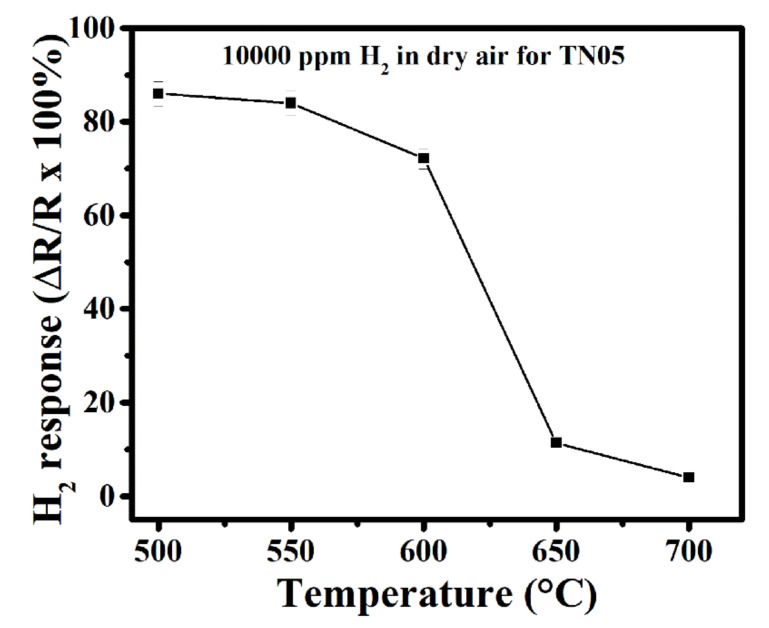
Response of gas sensor based on 0.5% Ni-doped TiO_2_ to 10,000 ppm of H_2_ in dry air at different operating temperatures.

In order to determine the effect of operating temperature on sensor response, the responses of TN05 towards 10,000 ppm H_2_ in dry air were measured from 500 to 700 °C. This concentration was chosen based on the shortest response time observed during the measurement. The sensor response decreases with increasing operating temperature as can be observed in Figure 7. It is well-known that the activation energy necessary for the reactions taking place during the sensing mechanism (oxidation of H_2_ in our case) depends on the operating temperature. However, the decrease in sensor response observed at operating temperatures above 550 °C can be explained by the Langmuir adsorption probability theory. In fact, above the operating temperature required for optimum activation energy, the desorption rate of H_2_ on the sensor surface becomes higher than its adsorption rate, leading to a decrease in gas sensitivity. We should mention that lower operating temperatures often result in longer sensor response times (at 500 °C tres = 64 s; 550 °C tres = 31 s; 600 °C tres = 23 s). Therefore, 600 °C seems to be the ideal operating temperature as the sensor response is still high enough, while a reasonably good response time is also observed. We therefore choose 600 °C to be the operating temperature in this work.

**Figure 8:**
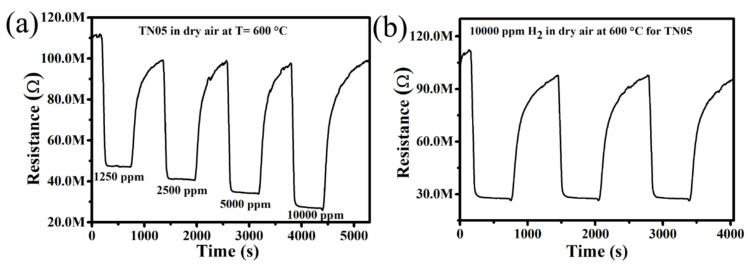
Dynamic response of gas sensors based on 0.5% Ni-doped TiO_2_ to (**a**) various concentrations of H_2_ and (**b**) 10,000 ppm H_2_ at 600 °C.

Figure 8a displays the dynamic response of the sensor TN05 at 600 °C towards various H_2_ concentrations (e.g., 1250, 2500, 5000 and 10,000 ppm) in synthetic dry air. As can be seen, upon gas exposure, the resistance value decreases until a quasi-equilibrium is reached. When the test gas flow is stopped, this resistance value returns without perfectly reaching its original baseline value. This sequence holds for all the applied H_2_ concentrations, illustrating acceptable stability of the sensor behavior and a very good reproducibility of the TN05 sensor. We should note that the drift observed in dry air is because the sensor requires more time until it is fully cleaned. The sensor responses obtained were 52, 61, 65, and 73% for 1250, 2500, 5000, and 10,000 ppm of H_2_, respectively. The observed increase in gas response with increasing H_2_ concentration suggests the possible use of the sensor for quantitative analysis, even though there is a slight drift due to the incomplete desorption of H_2_ molecules after the test gas is vented. In the frame of a preliminary study, to further verify whether this sensor is reliable and shows reproducible results at the same H_2_ concentration, the sensor tests were reproduced by injecting 10,000 ppm H_2_ in synthetic dry air into the test chamber under the same test conditions three times. According to Figure 8b, which shows the dynamic curve recorded, the average value of the gas response is 73%, with a slight insignificant variation. These short-term results may allow the conclusion that the H_2_ sensor based on TN05 yields a sensing capability of reasonably good reproducibility.

**Figure 9:**
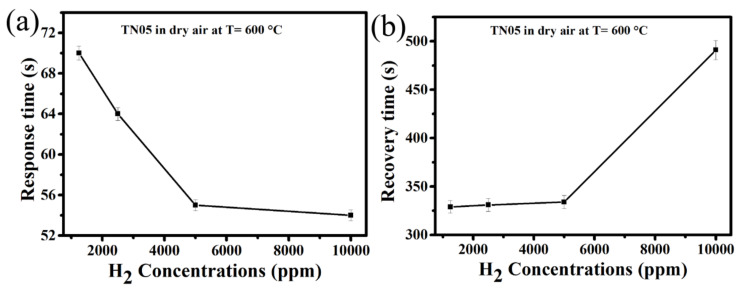
Relationship between H_2_ concentrations and (**a**) the response time (**b**) the recovery time for 0.5% Ni-doped TiO_2_ at 600 °C.

The response and recovery times of a sensor in real-time measurements are some of its most essential features. Therefore, we evaluated the response and recovery times of TN05 in dry synthetic air for different H_2_ concentrations (1250–10,000 ppm). The response and recovery times were determined by measuring the time that is needed for the gas sensor to complete 90% of the total resistance change in the cases of adsorption and desorption, respectively. The response time for all the sensors was measured when the resistance starts to change after introducing H_2_. The time that is required for the gas to reach and fill the test chamber fully is not considered. Figure 9a shows that when the H_2_ concentration increases from 1250 to 10,000 ppm, the response time decreases from 70 (1.1 min) to 54 s (less than one minute) while the recovery time (Figure 9b) increases from 329 (5.48 min) to 491 s (8.18 min), respectively. This observed trend in the response time is in accordance with what has been reported in the literature [40,41]. This can be explained by the basic effects of surface coverage kinetics and diffusion phenomena. The covering rate of the surface is low at low H_2_ concentration, resulting in a longer response time. In other words, more time is required to cover the entire surface at low concentration. At high H_2_ concentrations, the response time decreases as the rate of the surface coverage increases. The trends observed for the recovery time can be explained by the fact that, at lower concentrations, there will be less molecules attached to the surface. Thus, the required time for H_2_ to be desorbed will be lower than the time necessary for the desorption at high gas concentrations, where the amount of gas molecules attached to the surface will be comparably high. The morphology and homogeneity (i.e., homogeneous distribution of low Ni-dopant content (TN05) within the fine spherical particles)) achieved by the applied co-precipitation synthesis route is another factor for relatively shorter response times.

**Figure 10:**
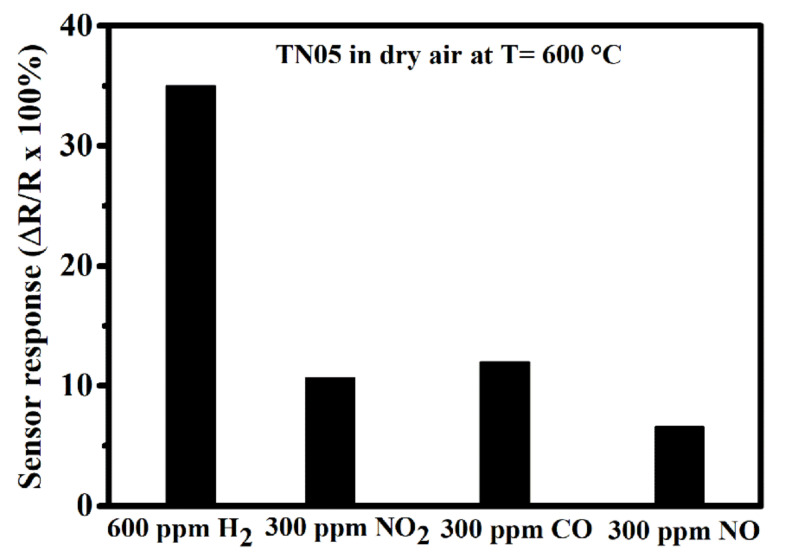
Response of gas sensor based on 0.5% Ni-doped TiO_2_ to various gases including 600 ppm of H_2_, 300 ppm of NO_2_, NO and CO all in dry air at 600 °C.

Good sensitivity and response rate are not the only determining factors for the suitability of a sensor. The achievement of a great selectivity towards the target gas is also a key parameter and a very important characteristic. Therefore, the responses of the TN05 gas sensor towards a variety of interference gases including NO_2_, CO, and NO at 600 °C in dry synthetic air were explored to evaluate its selectivity. As observed in Figure 10, the response of this sensor towards 600 ppm of H_2_ (35%) is at least a factor of two higher than that towards 300 ppm of CO (12%), 300 ppm of NO_2_ (11%), and 300 ppm of NO (7%). It should be noted that 600 ppm of H_2_ was the smallest concentration possible in our present set-up. Nevertheless, the sensor’s H_2_-response was a factor of two greater compared to that for 300 ppm of all the tested interfering gases, yielding the highest value and indicating a relatively high selectivity potential of the sensor towards H_2_.

In general, humidity has an effect on sensing performance, and this is more pronounced at room or low temperature, where water molecules can easily attach and dissociate on the material surface, reducing the active reaction sites. Our previous work, however, showed that at high temperature, the dissociation of water vapor occurs before reaching the material surface and sometimes has a positive effect on the sensing properties (e.g., increasing the selectivity) [40]. However, some examples in the literature report that adopting an orientation of TiO_2_ towards the (002) plane can greatly reduce the effect of humidity on the sensing performance [42]. Therefore, as a perspective of this work, a further study is planned in which sensing layers that contain (002) oriented 0.5% Ni-doped TiO_2_ particles are prepared by co-sputtering deposition, in order to determine the effect of such orientation on the sensing performance under humid conditions.

Table 3 highlights a comparison between the optimum sensing temperature achieved in the present sensor and the other doped TiO_2_ used for H_2_ detection, which were reported in the previous literature. It can be evidently observed that this present sensor operates at the highest temperature of 600 °C.

### Sensing Mechanism

**Figure 11:**
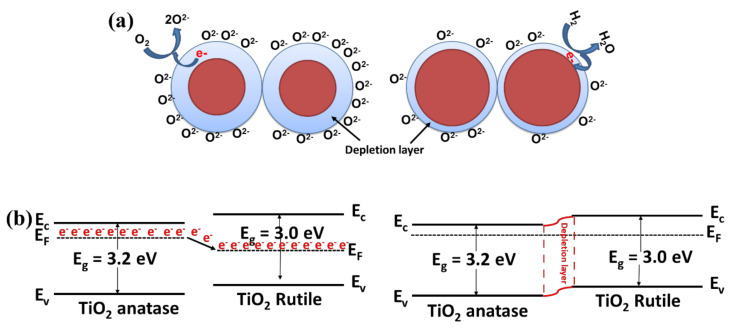
(**a**) An illustration of the general hydrogen sensing mechanism of TiO_2_-based sensors (**b**) energy band diagram of TiO_2_ anatase and rutile.

The gas-sensing mechanism of metal oxides is fundamentally a surface related issue resulting in a change in sensor resistance that is tightly related to the adsorption or desorption processes of target gases [47]. After the Ni-doped TiO_2_ nanostructure is exposed to air, oxygen molecules are adsorbed on the surface. Depending on the temperature, adsorbed oxygen ion species (O_2_^−^, O^−^, O^2−^) are formed by extraction of conduction band electrons and by generating a space charge conduction area (Equation (1)). For n-type semiconductors, this causes an increase in sensor resistance. When the target gas (hydrogen) is injected, it is adsorbed on the material surface and then gets oxidized (Equation (2)), resulting in the return of electrons back to the conduction band. As a consequence, this reduces the space charge width as well as the resistance. This loading of hydrogen leads to a large change in the depletion region of metal oxides (Figure 11a). As observed from the results, the gas sensing is highly influenced by Ni doping of TiO_2_. First, Ni doping into TiO_2_ nanostructures provides more active sites due to the creation of more oxygen vacancies (as revealed by Raman results in Figure 2) resulting from the substitution of Ti by Ni (with lower valence). This enhances chemisorption, and a larger change in sensor response is achieved in comparison to that for pristine TiO_2_. On the other hand, the sensing mechanism can be highly influenced by n–n junction formation due to the difference in bandgap energy between anatase and rutile [48]. The maximum number of junctions might have formed at the interface between anatase and rutile under the optimum conditions when the Ni concentration was approximately 0.5 mol.%. The charge transfer theory can explain the formation of the Schottky barrier at the anatase/rutile junction. In fact, at the interface of anatase and rutile, an inner electric field will be created, allowing electrons to flow from anatase (with higher band gap) to rutile, whereas the holes flow in the opposite direction until the Fermi level equilibrates. This generates an electron-depleted layer at the interface of anatase and rutile, which bends the energy band (Figure 11b). The creation of two depleted layers, one at the surface of the individual grains as a result of the adsorption of oxygen species, and the other at the interface of anatase and rutile, facilitates higher oxygen adsorption on the sensor surface, possibly yielding a higher concentration site reaction. Therefore, by interacting with the H_2_ gas, more electrons are released back to the conduction band, eventually leading to an enhanced response. Another hypothesis to explain the influence of n-n junctions is that the baseline resistance of the TN05 sample (where we have the highest number of n-n junctions) in air (R_air_) is higher than that of other samples (undoped TiO_2_: R_air_ = 89 MΩ, TN05: R_air_ = 116 MΩ, TN1: R_air_ = 77 MΩ and TN2: R_air_ = 70 MΩ) at 600 °C, as observed in Figure 5. This is due to the fact that at the contact points between anatase and rutile, a depletion layer occurs that increases the contact resistance between the grains, which in the present case, dominates the overall resistance of the layer. A similar trend has already been reported in the literature [38,39,49]. When H_2_ is introduced, the electrons that are released through its oxidation increase the electron concentration. This results in a large decrease in the resistance, and thus increases the sensor response compared to the situation without junctions or with fewer junctions.

O_2_ (gas) + 4e^−^ → 2O^2−^ (ads) (1)

2H_2_ (g) + 2O^2−^ (ads) → 2H_2_O (g) + 4e^−^ (2)

## 4. Conclusions

Pristine TiO_2_ and TiO_2_ doped with different amounts of Ni (0.5, 1 and 2%) have been successfully synthesized by a simple oxalate co-precipitation route. Their hydrogen sensing properties at high temperatures (600 °C) were investigated. According to the XRD results, a mixture of anatase and rutile polymorphs were obtained with the amount of rutile increasing with increasing nickel concentration. The 0.5% Ni-doped TiO_2_ contains almost equal amounts of anatase and rutile. Enhanced sensing properties with respect to H_2_ were observed for 0.5% Ni-doped TiO_2_ in comparison to undoped and 1 and 2% Ni-doped TiO_2._ A good sensor response of 72%, the best response rate of 1.2 s^−1^, and a good selectivity for H_2_ over NO_2_, NO and CO were obtained. The highest number of n-n junctions between anatase and rutile polymorphs was probably the principal factor responsible for the enhanced gas sensing performance of 0.5% Ni-doped TiO_2_. These results certainly open up a new opportunity for manufacturing gas sensors for high-temperature H_2_ detection using titanium-based materials synthesized by co-precipitation.

## Figures and Tables

**Table 1:** Lattice parameters, estimated phase composition and Brunauer–Emmet–Teller (BET) values of the co-precipitation synthesized undoped and all Ni-doped TiO_2_ powders.

Sample Name	Lattice Parameter of Anatase, *a* (Å)	Lattice Parameter of Rutile, *a* (Å)	Phase Composition (%)	BET (m^2^/g)
TN0	3.7874 ± 0.0001	/	Anatase (98)/Rutile (2)	7.8
TN05	3.7839 ± 0.0001	4.5932 ± 0.0002	Anatase (53)/Rutile (47)	6.9
TN1	3.7836 ± 0.0002	4.5928 ± 0.0002	Anatase (21)/Rutile (79)	6.4
TN2	/	4.5939 ± 0.0002	Anatase (1)/Rutile (99)	10.7

**Table 2:** EDX results for TN0, TN05, TN1 and TN2.

Samples	Ti (at.%)	Ni (at.%)	O (at.%)	Ni/Ti × 100 (%) Experimental	Ni/Ti × 100 (%) Expected
TN0	33.25	/	66.85	0	0
TN05	33.12	0.16	66.73	0.48	0.5
TN1	24.62	0.24	75.14	0.97	1
TN2	21.76	0.43	77.81	1.98	2

**Table 3:** Comparison of the optimum sensing temperature of the present sensor and that of devices previously reported in the literature.

Material	Synthesis Method	Temperature (°C)	Gas Concentration (ppm)	Sensor Response	Reference
Ni-doped TiO_2_ nanotube	Anodic oxidation	200	1000	13%	[43]
Ni-doped TiO_2_ nanotube	Electrochemical anodization	200	5000	60%	[25]
Cr-doped TiO_2_ spherical	Flame spray synthesis	350	3000	50%	[44]
Nb-doped TiO_2_ nanotube	anodization	RT	1000	30%	[45]
Cr doped TiO_2_ irregular morphology	Sol-gel	500	1000	99.8%	[15]
Al-doped TiO_2_	Sputtering	350	2500	99.2%	[24]
Al-V doped TiO_2_ Nanotube	Electrochemical anodization	300	1000	50%	[46]
Nb-doped TiO_2_ nanorod	Hydrothermal	RT	8000	98.9%	[26]
Ni-doped TiO_2_ nanoparticles	Co-precipitation	600	5000	65%	**This work**
